# Scaffold-free cell-based tissue engineering therapies: advances, shortfalls and forecast

**DOI:** 10.1038/s41536-021-00133-3

**Published:** 2021-03-29

**Authors:** Andrea De Pieri, Yury Rochev, Dimitrios I. Zeugolis

**Affiliations:** 1 Regenerative, Modular & Developmental Engineering Laboratory (REMODEL), Biomedical Sciences Building, National University of Ireland Galway (NUI Galway), Galway, Ireland; 2 Science Foundation Ireland (SFI) Centre for Research in Medical Devices (CÚRAM), Biomedical Sciences Building, National University of Ireland Galway (NUI Galway), Galway, Ireland; 3 Proxy Biomedical Ltd., Coilleach, Spiddal, Galway, Ireland; 4 Regenerative, Modular & Developmental Engineering Laboratory (REMODEL), Faculty of Biomedical Sciences, Università della Svizzera Italiana (USI), Lugano, Switzerland

**Keywords:** Regenerative medicine, Tissue engineering, Cell delivery

## Abstract

Cell-based scaffold-free therapies seek to develop in vitro organotypic three-dimensional (3D) tissue-like surrogates, capitalising upon the inherent capacity of cells to create tissues with efficiency and sophistication that is still unparalleled by human-made devices. Although automation systems have been realised and (some) success stories have been witnessed over the years in clinical and commercial arenas, in vitro organogenesis is far from becoming a standard way of care. This limited technology transfer is largely attributed to scalability-associated costs, considering that the development of a borderline 3D implantable device requires very high number of functional cells and prolonged ex vivo culture periods. Herein, we critically discuss advancements and shortfalls of scaffold-free cell-based tissue engineering strategies, along with pioneering concepts that have the potential to transform regenerative and reparative medicine.

## Introduction

Cell-based therapy has gained tremendous interest in the past decades and holds promise for transforming treatments for a wide range of injuries and diseases. The market for cell therapy products is set to expand based on increasing investment from the industry and the implementation of advanced manufacturing technologies. In fact, the global cell therapy market is forecast to reach €7.24 billion by 2025 with compound annual growth rate of 14.9% from 2017, which would make it the fastest growing sector in the regenerative medicine industry^1^.

**Fig. 1:**
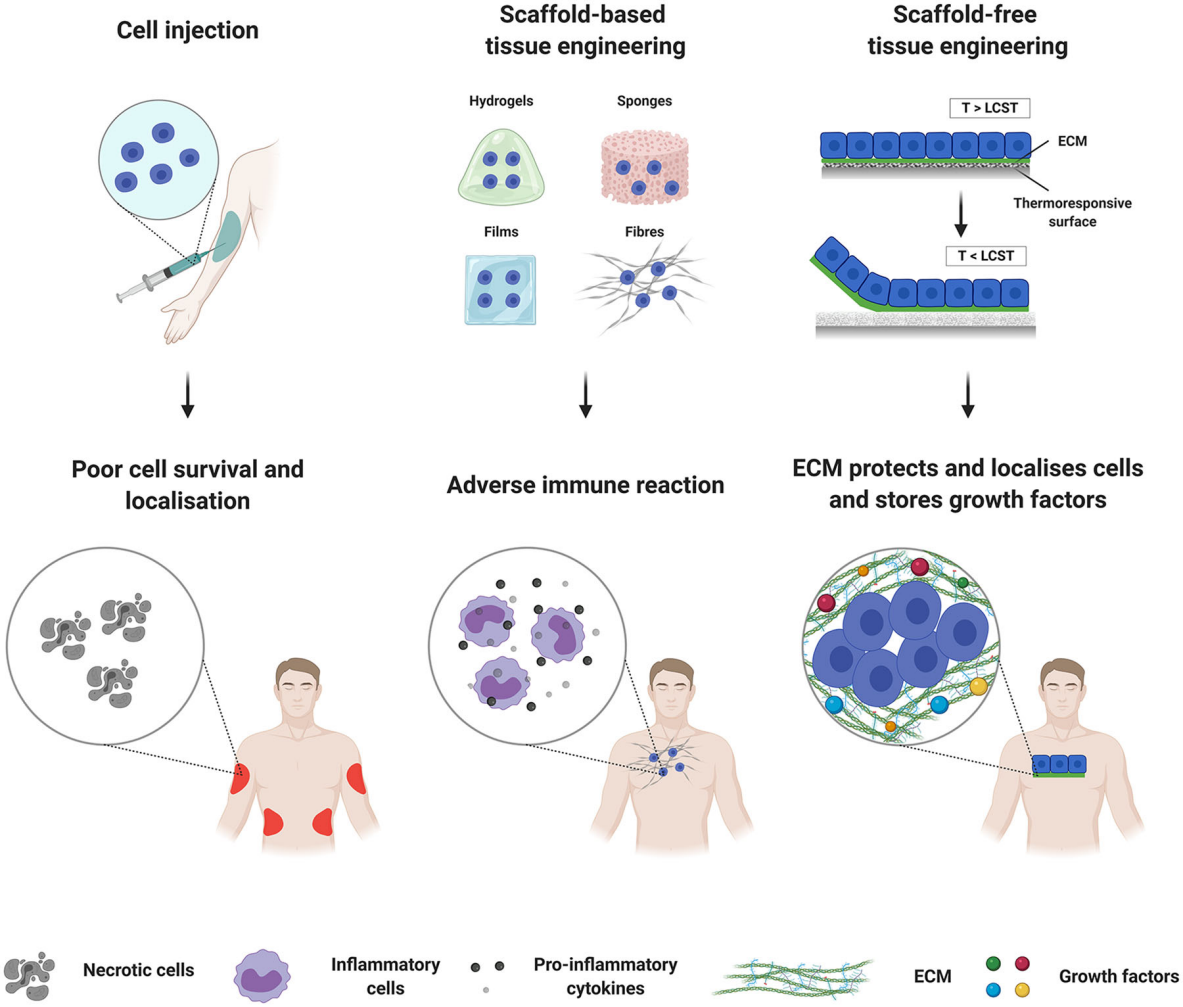
Cell-based tissue engineering therapies. The figure was created with BioRender.com.

Cells have enormous therapeutic potential, as they provide sophisticated tissue-specific mechanisms of actions that chemical compounds cannot imitate. Mesenchymal stem cells (MSCs), permanently differentiated cells and, more recently, induced pluripotent stem cells (iPSCs) have been used in preclinical and clinical trials with successful outcomes^2–7^. One critical aspect for therapeutic efficacy after cell transplantation is the delivery method (Fig. 1). The optimal cell delivery format should ensure high cell retention and survival rate, good tissue integration and zero to low side effects for the patient. Intra-venous/intra-arterial infusion or direct intra-tissue injection are the most common routes of cell transplantation. However, these approaches have shown limited success, mainly due to the poor cell localisation, retention and survival at the site of injury post-transplantation. In fact, numerous studies have shown that <5% of the injected cells persist at the site of injection in the first day(s) after transplantation, indicating a survival rate of as low as 1% (refs. ^8–12^).

Scaffold-based tissue engineering was pioneered to overcome the limitations of direct cell suspensions, aiming not only to develop efficient cell delivery strategies, but also to produce elegant three-dimensional (3D) tissue analogues. Traditional scaffold-based tissue engineering strategies employ a cytocompatible, biodegradable and mechanically stable natural or synthetic in origin polymeric scaffold with a fully interconnected porous network for efficient transport and exchange of oxygen, nutrients and metabolites^13,14^. Although very many scaffold conformations (e.g., hydrogels^15,16^, sponges^17,18^, fibres^19,20^, films^21,22^) have been developed, and have demonstrated safety and efficacy in preclinical setting and phase I clinical trials as cell delivery vehicles, only a handful of them constitute a Food and Drug Administration (FDA)/European Medicines Agency (EMA) approved device (Table 1). This limited technology transfer from laboratory benchtop to clinical applicability has been attributed to component (e.g., limited understanding of the mechanism of action of the various device components; device components do not comply with regulatory frameworks; toxicity issues) and process (e.g., too complex to allow for large-scale efficient and reproducible manufacturing; too long to be profitable) limitations.

**Table 1:** Commercially available scaffold-based cell systems.

Clinical indication	Product, manufacturer	Technology description
Skin	Apligraf^®^, Organogenesis (USA)	Bilayer skin equivalent consisting of bovine type I collagen matrix cultured with allogeneic neonatal DF and EK licensed for the treatment of diabetic foot and venous leg ulcers
	Dermagraft^®^, Organogenesis (USA)	Dermal substitute consisting of bioabsorbable polyglactin mesh cultured with neonatal allogenic DFs licensed for the treatment of diabetic foot ulcers
	DenovoDerm^™^, Cutiss (Switzerland)	Dermal substitute consisting of bovine collagen type I cultured with autologous DFs, currently under phase II clinical trials for the treatment of burns
	DenovoSkin^™^, Cutiss (Switzerland)	Bilayer skin equivalent consisting of bovine collagen type I hydrogel cultured with autologous DFs and EKs, currently under phase II clinical trials for the treatment of burns
	OrCel^®^, Ortec International (USA)	Bilayer skin equivalent consisting bovine collagen type I sponge cultured with allogeneic DFs and EKs licensed for the treatments of burns
	TransCyte^®^, Advanced BioHealing (USA)	Dermal substitute consisting of an absorbable polyglycolic acid mesh cultured with allogenic neonatal DFs licensed for the treatment of burns
Cartilage	Biocart^™^ II, Histogenics Corporation (USA)	Fibrin and hyaluronic acid-based scaffold seeded with autologous CCs under phase II clinical trials for the treatment of cartilage lesions of the knee
	BioSeed^®^-C, Biotissue Technologies (Switzerland)	Fibrin, polyglycolic/polylactic acid and polydioxanone-based scaffold seeded with autologous CCs licensed for the treatment of articular cartilage injuries
	CaReS^®^, Arthro Kinetics, Germany	Rat type I collagen hydrogel seeded with autologous CCs licensed for the treatment of articular cartilage injury
	MACI^®^, Vericel (USA)	Collagen I/III scaffold from porcine peritoneum seeded with autologous CCs licensed for the treatment of cartilage lesions of the knee
	NeoCart^®^, Histogenics Corporation (USA)	Porous bovine type I collagen scaffold seeded with autologous CCs under phase III clinical trials for the treatment of articular cartilage lesions
	NOVOCART^®^ 3D, B. Braun-Tetec (Germany)	Bilayer type I collagen sponge containing chondroitin sulfate seeded with autologous CCs under phase III clinical trials for the treatment of articular cartilage lesions
Bone	BIO4^®^, Osiris Therapeutics (USA)	Bone matrix seeded with allogenic MSCs osteoprogenitor cells and osteoblasts licensed as bone allograft
	Osteocel^®^ Plus, NuVasive (USA)	Bone matrix seeded with allogenic MSCs licensed as bone grafting after lateral interbody fusion
	Trinity Elite^®^, Orthofix (USA)	Cancellous bone matrix seeded with allogenic MSCs and osteoprogenitor cells licensed as bone allograft
	ViBone^®^, AZIYO Biologics, (USA)	Cancellous bone matrix seeded with allogenic MSCs and osteoprogenitor cells licensed as bone allograft
	ViviGen^®^, Johnson & Johnson (USA)	Cortico-cancellous bone matrix seeded with allogenic osteoprogenitor cells licensed as bone allograft
Cornea	NT‑501, Neurotech (USA)	Hollow polyether-sulfone fibres seeded with genetically engineered retinal pigment epithelial cell line under phase III clinical trials for the treatment of retinal degenerative diseases
Blood vessels	VascuGel^™^, Pervasis (USA)	Gelfoam^®^ gelatin matrix seeded with allogenic human aortic endothelial cells under phase III clinical trials for the treatment of peripheral artery disease
Oesophagus	Cellspan^®^, Biostage (USA)	Biostage^™^ polyurethane scaffolds seeded with autologous ADSCs licensed for the treatment of oesophageal injuries

*ADSCs* adipose-derived stem cells, *CCs* chondrocytes, *DFs* dermal fibroblasts, *EKs* epidermal keratinocytes, *MSCs* mesenchymal stem cells, *iPSCs* induced pluripotent stem cells.

Considering that tissues are formed by cells and their secreted components with precision, efficiency, order and sophistication that is still unmatched by human-made devices, it made sense to develop means to exploit this inherent capacity of cells for the development of tissue analogues. In this case, the cell-secreted extracellular matrix (ECM) acts as carrier and protector of the transplanted cells. Further, as no artificial scaffold is used, the produced constructs are of superior biocompatibility and with less chances of foreign body response than any other technology that has been assessed to-date. Although the scaffold-free tissue engineering concept is far from new (the first scaffold-free device was developed in 1975 (ref. ^23^), assessed in preclinical models in 1980 (ref. ^24^) and assessed in humans in 1981 (ref. ^25^)), only a handful of products have been commercialised (Table 2). Herein, we critically discuss recent advancements and limitations that prohibit wide acceptance, clinical translation and commercialisation of scaffold-free cell-based tissue engineering strategies.

**Table 2:** Commercially available scaffold-free cell systems.

Clinical indication	Product, manufacturer	Technology description
Skin	Epicel^®^, Vericel Corporation (USA)	Autologous EK sheets licensed for the treatment of burns
Cartilage	Chondrosphere^®^, CO.DON AG (Germany)	Autologous 3D CCs spheroids under phase III clinical trials for the treatment of knee articular cartilage injuries
	RevaFlex^™^, ISTO Technologies (USA)	Allogeneic juvenile CC sheets under phase II clinical trials for the treatment of articular cartilage injury
	CellSeed Inc (Japan)	Allogenic CC sheets under clinical trials for the treatment of cartilage defects and knee osteoarthritis
Cornea	Holoclar^®^, Chiesi Farmaceutici (Italy)	Autologous epithelial corneal cell sheet licensed for the treatment of limbal stem cells deficiency
Heart	HeartSheet^®^, Terumo (Japan)	Autologous skeletal myoblast sheets licensed for the treatment of severe heart failure caused by chronic ischaemic heart disease
Blood vessel	LifeLine^™^, Cytograft (USA)	Autologous fibroblasts tubular constructs licensed as shunts for haemodialysis
Esophagus	CellSeed Inc (Japan)	Autologous oral epithelial cell sheets licensed for the treatment of oesophageal ulcers after endoscopic surgery for oesophageal cancer

*CCs* chondrocytes, *EK* epidermal keratinocytes.

## Cell sheet tissue engineering

Tissues and organs are comprised of different cell types that are surrounded by their secreted, tissue-specific ECM. This densely populated microenvironment allows for efficient cell–cell and cell–ECM communications, which determine cell fate and function^26,27^. Cell sheet tissue engineering takes advantage of the close cell–cell and cel–ECM interactions to autonomously engineer microtissues, utilising the temperature-responsive cell culture technology. The temperature-responsive polymer used [usually poly(N-isopropylacrylamide) (pNIPAM), although a variety of polymers with diverse properties have been developed and assessed over the years (Table 3)] undergoes a transition from hydrophobic to hydrophilic across its lower critical solution temperature (LCST) of 32 °C. At temperatures >32 °C, the surfaces are hydrophobic and allow for the culture of adherent cells as on normal tissue culture polystyrene at 37 °C. As the cells grow, they deposit ECM proteins that assemble into interconnected tissue-like structures. When the temperature is reduced <32 °C, pNIPAm molecules become highly hydrated, thus the pNIPAM grafted surfaces become hydrophilic. After this thermal transition, cultured cells almost spontaneously detach from the pNIPAm surface as a contiguous cell sheet with preserved cell–cell junctions and deposited ECM^28,29^. Since ECM proteins remain on the surface of the cell sheets, they are adhesive to biological surfaces and therefore can be transplanted to injured tissues without the need of sutures or external fixation^30^. The deposited ECM also acts as a depot of numerous bioactive and trophic molecules, and also protects and localises the transplanted cells at the site of implantation^31–33^. This unique approach, with or without the use of a temperature-responsive polymer, has been used to develop implantable devices out of numerous human cells, and their safety and efficacy has been demonstrated in preclinical and clinical setting for a diverse range of clinical indications (Table 4).

**Table 3:** Temperature-responsive polymers that have been used in the development of scaffold-free cell sheet.

Polymer	LCST	Contact angle	Cells	Detachment time (min)	Refs.
Elastin-like recombinamers	21-32 °C	<LCST, 58–67° >LCST, 65–76°	Human DFs Human ADSCs	20	^190^
HBC	29 °C	<LCST, 30° >LCST, 17°	Rat smooth muscle cell Human MSC Human disc cells	30–60	^35,191,192^
MC	32 °C	NA	3T3 mouse fibroblasts Human ADSCs	20–30 2–3	^193,194^
PIPOx	36 °C	<LCST, 61–62° >LCST, 72–73°	Human DFs	30	^195,196^
PNAAMe P(NAAMe-co-NAGMe)	17 °C	<LCST, 142–146° >LCST, 136–140°	3T3 mouse fibroblasts	1	^197^
PNVCL	31-32 °C	<LCST, 25° >LCST, 70°	3T3 mouse fibroblasts	30	^198^
PVME	32-34 °C	NA	Human corneal endothelial cell	60	^199^
PVME-PVMEMA	33 °C	NA	Human corneal endothelial cell	60	^200^
PEO-PPO-PEO (Pluronic^®^ F-127)	30 °C	<LCST, 21–11° >LCST, 41–48°	L929 mouse fibroblasts	NA	^201,202^

*ADSCs* adipose-derived stem cells, *DFs* dermal fibroblasts, *HBC* hydroxybutyl chitosan, *LCST* low critical solution temperature, *MC* methylcellulose, *NAGMe* glycine methyl ester-based acrylamide, *PEO* poly(ethylene oxide), *PIPOx* poly(2-isopropyl-2-oxazoline), *PNAAMe* alanine methyl ester-based polyacrylamide, *PNVCL* poly(N-vinylcaprolactam), *PPO* poly(propylene oxide), *PVME* poly(vinyl methyl ether), *PVMEMA* poly (vinyl methyl ether-maleic acid).

**Table 4:** Indicative examples of successful stories of human scaffold-free cell systems in preclinical and clinical setting.

Clinical indication	Technology description	Preclinical/clinical outcome	Ref.
Skin	Three layers of human ADSCs sheets cultured on temperature-responsive dishes	Transplantation into mice with full-thickness wounds promoted neovascularisation, the regeneration of thicker epidermis and the formation of new hair follicles 21 days post implantation	^67^
	Three cellular constructs composed of human EKs, DF and DMECs cultured on temperature-responsive dishes	Transplantation into mice with full-thickness wounds showed that cells were engrafted into the host wound bed and were present in the neotissue formed up to 14 days post implantation. The 3D constructs significantly contributed to re-epithelialisation and neovascularisation	^203^
Cartilage	Three layers of a of autologous human CCs co-cultured with synovial cells on temperature-responsive dishes	Clinical trial on eight patients affected by knee osteoarthritis. Cell sheets promoted hyaline cartilage regeneration 36 months postoperatively	^204^
Bone	Human DPSCs cultured on temperature-responsive dishes, differentiated towards the osteogenic lineage with a helioxanthin derivative	Transplantation into mouse calvaria defects demonstrated that DPSC sheets treated with helioxanthin derivative-induced bone regeneration more extensively than the control sheets 8 weeks after transplantation	^205^
Cornea	Human autologous oral mucosal epithelial cells cultured on temperature-responsive dishes with 3T3 feeder cells that had been treated with mitomycin C	Clinical trial on four patients affected by total limbal deficiency. One week after cell transplantation complete re-epithelialisation of the corneal surfaces occurred. Corneal transparency was restored and postoperative visual acuity improved remarkably. During a mean follow-up period of 14 months, all corneal surfaces remained transparent	^206^
Heart	Human autologous skeletal stem cell sheets cultured on temperature-responsive dishes	Clinical trial on 15 ischaemic cardiomyopathy patients and 12 patients with dilated cardiomyopathy. Cell sheet implantation improved ischaemic cardiomyopathy patients’ exercise capacity, and symptoms. Limited efficacy was observed in dilated cardiomyopathy patients	^207^
	Human BMSCs sheets cultured on temperature-responsive culture dishes	Transplantation over the infarct myocardium of porcine ischaemic cardiomyopathy models attenuated left ventricular remodelling and improved cardiac function 8 weeks after implantation	^208^
	Three layers of human iPSCs differentiated into cardiovascular cell populations (cardiomyocytes, endothelial cells and vascular mural cells) cultured on temperature-responsive culture dishes	Transplantation over the infarcted hearts of athymic nude rats significantly improved cardiac function and neovascularisation 8 weeks after transplantation	^209^
Oesophagus	Human oral mucosal epithelium cells cultured on temperature-responsive culture dishes	Clinical trial on nine patients who underwent oesophageal endoscopic submucosal dissection to remove superficial oesophageal neoplasms. Cell sheet implantation induced complete re-epithelialisation occurred within 3–4 weeks, and no patients experienced dysphagia, stricture or other complications following the procedure	^210^
Periodontal ligament	Three layers of human autologous periodontal ligament-derived cell sheets, cultured with media containing autologous serum until reaching confluence on temperature-responsive culture dishes	Clinical trial on ten patients affected by periodontitis. Six months after the transplantation, reduction of periodontal probing depth, clinical attachment gain and increase of radiographic bone height, were improved	^211^
Liver	Three layers of hepatic cell sheets differentiated from human BMSCs treated with hexachrolophene and cultured on temperature-responsive culture dishes	Transplantation into non-obese diabetic severe immunodeficient mice with acute liver injury, suppressed the injury, enhanced regeneration and improved survival rates of the mice	^212^
Facial nerve injury	Human DPSCs were plated in six-well plates at 200,000 cells per well in media comprised among others of 20% foetal bovine serum and 5 ng/ml fibroblast growth factor 2 for 10–12 days.	Transplantation into immunocompromised rats with crushed buccal branch of the facial nerve. The cell sheet was wrapped around the injury. The cell sheets maintained nerve structure, accelerated axon regeneration and extension and enhanced electrophysiological functionality, following nerve injury.	^213^

*ADSCs* adipose-derived stem cells, *BMSCs* bone marrow stem cells, *CCs* chondrocytes, *DFs* dermal fibroblasts, *DMECs* dermal microvascular endothelial cells, *DPSCs* dental pulp stem cells, *EKs* epidermal keratinocytes, *iPSCs* induced pluripotent stem cells.

Over the years, significant strides have been achieved in developing tissue analogues, with high levels of architectural biomimicry^34^. For example, recapitulation of native anisotropic tissue topographies has been realised via the use of bi-directionally aligned temperature-responsive electrospun scaffolds^35^, microcontact printing of aligned fibronectin patterns^36,37^, photolithography on non-cell adhesive anisotropic patterns^38–40^, grafting of temperature-responsive polymers onto micropatterned poly(dimethysiloxane) substrates^41,42^ or unidirectional mechanical stimulation^43^. With respect to the development of 3D tissue-like assemblies, multi-layered cell sheet stacking has been proposed, which has resulted in the formation of sophisticated microtissues in vitro (e.g., skeletal muscle-like tissue out of myoblasts^44,45^, myocardial-like tissue out of cardiomyocytes^46^, annulus fibrosus-like tissue out of bone marrow MSCs^47^, tubular neural-like tissue out of astrocytes and iPSC-derived neurons^48^).

To further de-risk the technology and increase its scalability, automated systems have also been developed. For example, automated technologies have been utilised for the production of multi-layered tissue constructs, using robotic systems. Specifically, five layers of human skeletal muscle myoblast sheets were successfully stacked by a robotic apparatus within 100 minu, representing a cost-effective manufacturing system for the manipulation of cell sheets^49^. Automated modular platforms have also been assembled for the sequential seeding, expansion and cell (e.g., skeletal myoblasts, articular chondrocytes and iPSCs) sheet preparation that have been shown to maintain aseptic conditions and to produce high quality cellular constructs, comparable to those produced in manual operations^50^. Automated systems have been further advanced for the production of high number of cell sheet batches. For instance, 10 human oral mucosa epithelial cell sheets were simultaneously cultured into 5 separate fully closed culture vessels, automated with a circuit system, that yielded up to 50 cell sheets and satisfied the quality standards of manual procedures^51^.

Considering that the thickness limitation of 3D constructs without vascular networks is ~40–80 μm (ref. ^52^), co-culture cell sheet approaches have been proposed for the development of pre-vascularised networks^53,54^. For example, human umbilical vein endothelial cells co-cultured within human myoblasts sheets formed capillary-like structures within the construct and increased neovascularisation and graft survival after transplantation into the subcutaneous tissues of nude rats^55^. However, in order to support the long-term culture of thick 3D tissue equivalents, the formation of functional mature blood vessels is required. To this end, bioreactor systems have been utilised in combination with engineered vascular beds, based on resected femoral muscles or synthetic collagen gels, which allowed continuous perfusion of culture media, formation of a functional vasculature and survival of 12-layer cell sheets^56,57^.

For the development of tubular tissues, such as blood vessels^58^, tendons^59^ and neurons^48^, cell and deposited ECM layers are rolled into tubular structures. Automated systems have also been designed, which utilise a wrapping device module composed of a fibrin tube holder and a sliding dish, capable of rolling three layers of cardiomyocyte sheets without any deformation^60^. A pioneering study reported the fabrication of human biological tissue-engineered blood vessel composed of smooth muscle cells and skin fibroblast sheets, which were mechanically peeled off from the culture dishes and rolled onto a supportive tubular mandrel (polytetrafluoroethylene)^58^. After maturation (8 weeks under dynamic conditions) of the construct into a cohesive vascular structure, the supportive mandrel was removed and endothelial cells were seeded in the lumen, ultimately creating a well‐defined, three‐layered device with abundant ECM. The major limitation of this technology was the time required to produce functional grafts (~28 weeks^61^), which resulted in the company closing down. Advanced anisotropic tubular assemblies have also been realised, using surface patterning to culture human astrocytes and human iPSC‐derived neurons, to create neural microtissues^48^.

## Limitations and way forward

Despite the significant advances that have been achieved in the field of scaffold-free tissue engineering, the technology is far for optimal, as evidenced by the limited number of clinically and commercially available products. Several interconnected and interdependent limitations must be addressed for this technology to become clinical standard.

### Transitioning from 2D to 3D systems

**Fig. 2:**
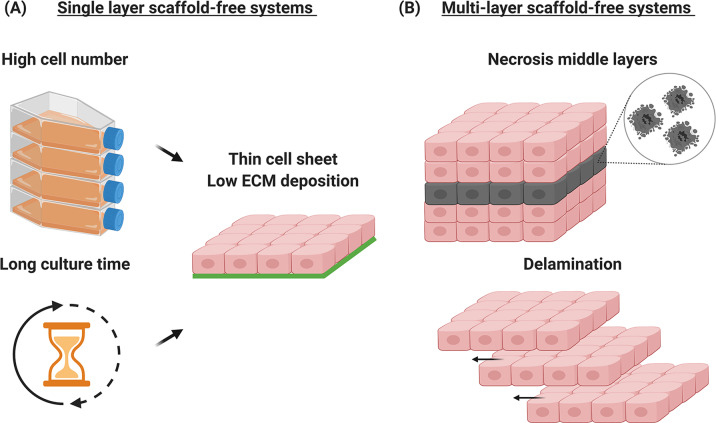
Limitations of scaffold-free tissue engineering. Traditional single-layer scaffold-free systems require high cell numbers and prolonged culture periods to produce barely 3D tissue equivalents (**A**). Due to the absence of sufficient ECM, cell necrosis and delamination frequently occur in multi-layer scaffold-free systems (**B**). The figure was created with BioRender.com.

The limiting factor in clinical translation and commercialisation of scaffold-free concepts is the high number of cells required to produce in a commercially relevant timeframe an ECM-rich and truly 3D tissue equivalent (Fig. 2A). Indeed, temperature-responsive surface derived single-layer cell sheets, as well as more elegant micro-stereolithography and electrochemical desorption for cell transfer systems^62,63^, require a substantial cell number and/or culture time to produce a barely 3D scaffold-free construct (Table 5). For example 104,000 cells/cm^2^ human endometrial gland-derived MSCs produced a 50 µm thick cell sheet after 5 days^64^, 300,000/cm^2^ iPSCs-derived cardiomyocytes produced a 10 µm thick cell sheet after 27 days^65^, 612,000/cm^2^ human corneal endothelial cells produced a 15 µm thick cell sheet after 28 days^66^. To overcome these limitations, multi-layer cell sheet stacking has been proposed. Regrettably, even this approach has been found wanting, as again very high cell numbers and relatively long culture times are required to develop borderline 3D implantable devices (Table 6). For instance, five layers of 1,000,000/cm^2^ human skeletal muscle myoblasts grown on a temperature-responsive dish for 5 days produced a 50 μm thick device^49^, three layers of 300,000/cm^2^ human adipose-derived MSCs grown on a temperature-responsive dish for 5 days produced a 20 μm thick device^67^, nine layers of 200,000/cm^2^ human iPSCs-derived cardiomyocytes per layer grown on a temperature-responsive dish for 7–10 days produced a 359 μm thick device^68^. Unfortunately, due to the absence of sufficient ECM, these high-density cultures are associated with poor nutrient and oxygen diffusion and waste accumulation in the middle layers that ultimately lead to cell necrosis^69,70^ and delamination^71^ (Fig. 2B). To address these issues, multiple operations of up to three layers have been proposed^52^; however, these multiple operations are associated with prolonged patient distress and high healthcare expenditure. To overcome the dimensionality limitation of scaffold-free systems, cell sheets (whole or in fragments) have been combined with various scaffold conformations (e.g., hydrogels^72^, particles^73^, tissue grafts^74^)/fabrication systems (e.g., electrospinning^75,76^, 3D printing^77,78^), which have been summarised elsewhere recently^79^.

**Table 5:** Single layer scaffold-free systems derived from human cells.

Cells	Cell density (cells/cm^2^)	Thickness (µm)	Culture time (days)	Ref.
Human nasal mucosal epithelial cells	50,000	25	12	^214^
Human iPSCs-derived cardiovascular cells	68,000	50	4	^209^
Human oral mucosal epithelial cells	80,000	50	14	^215^
Human endometrial-derived mesenchymal cells	100,000	20	4	^70^
Human ADSCs-derived cardiomyoblasts	104,000	20	4	^216^
Human endometrial gland-derived MSC	104,000	50	5	^64^
Human iPSCs-derived cardiomyocytes	300,000	10	27	^65^
Human corneal endothelial cells	612,000	15	28	^66^

*ADSCs* adipose-derived stem cells, *MSCs* mesenchymal stem cells, *iPSCs* induced pluripotent stem cells.

**Table 6:** Multi-layer scaffold-free systems derived from human cells.

Cells	Number of layers	Cell density (cells/cm^2^/layer)	Thickness (µm)	Culture time (days)	Ref.
Human chondrocytes	3	50,000	100	25	^204^
Human endometrial-derived mesenchymal cells	4	100,000	60	11	^70^
Human iPSCs-derived cardiomyocytes	9	200,000	359	7–10	^68^
Human ADSCs	3	300,000	20	5	^67^
Human skeletal muscle myoblasts	5	1,000,000	50	5	^49^

*ADSCs* adipose-derived stem cells, *iPSCs* induced pluripotent stem cells.

### Hypoxia

Considering the importance of ECM in regulating cell survival and tissue homoeostasis^80,81^, it is imperative to develop means to accelerate ECM synthesis and deposition. Physiological hypoxia (<10% O_2_) poses a biochemical cue crucial for the regulation of ECM synthesis and deposition. Indeed, hypoxia has been shown to increase mRNA levels of procollagen α1(I) in fibroblasts isolated from different tissues^82–85^. Hypoxia also regulates ECM homoeostasis through the activation of hypoxia-inducible transcription factor 1 (HIF-1)^86^. HIF-1 regulates collagen secretion and deposition by driving the transcription of prolyl 4-hydroxylase, which catalyses intracellularly the hydroxylation of proline and lysine residues and lysyl oxidase, which catalyses extracellularly collagen crosslinking^87–90^. Hypoxia therefore can be an ally in the fabrication of biomimetic tissue-engineered constructs.

Considerable efforts have been conducted to optimise oxygen levels of cultured cells, in order to control (stem) cell fate^91^ and promote ECM synthesis and deposition for the desired tissue engineering application (Table 7), such as skin^92^, cartilage^93,94^, bone^95,96^, tendon^97,98^ and heart^89^. For example, low oxygen tension (5% O_2_) has been shown to retain undifferentiated and multipotent status of MSC cultures^99,100^. In addition, hypoxia modulates the paracrine activity of MSCs and enhances the secretion of soluble growth factors, especially pro-angiogenic factors, such as vascular endothelial growth factor (VEGF)^101^. In scaffold-free tissue engineering, only few studies have utilised low oxygen tension for the production of implantable cell constructs. For example, multi-layered human chondrocyte sheets fabricated in a co‐culture system with synoviocytes and cultured at 2% oxygen tension showed greater cell metabolic activity and proliferation compared to cells cultured at 21% oxygen tension. Furthermore, hypoxic conditions accelerated and enhanced the deposition of cartilage-specific ECM, mainly composed of proteoglycans and collagen type II (ref. ^102^). Preconditioning of rabbit BMSCs or mouse cardiosphere-derived cell sheets under 2% oxygen tension, remarkably increased the expression of VEGF and significantly improved left ventricular function in myocardial infarction models in comparison to cells cultured under normoxia condition^103,104^.

**Table 7:** Influence of hypoxia on ECM synthesis and deposition in vitro.

Clinical indication	Cells	Oxygen tension (%)	Outcome	Ref.
Skin	Human DFs Human EKs	3	Improved epidermal morphogenesis and barrier formation in engineered human skin equivalents	^92^
Cartilage	Human BMSCs	5	Increased gene (Sox transcription factors) and protein (collagen type II, aggrecan) expression of chondrogenic markers	^93^
	Human CCs	2	Increased gene expression of collagen type II and aggrecan, and deposition of sulfated glycosaminoglycan	^94^
Bone	Human BMSCs	5	Increased gene expression of osteogenic and angiogenic markers, such as alkaline phosphatase, osteocalcin and VEGF	^96^
	Human ADSCs	3	Increased gene expression of collagen type I and alkaline phosphatase, and promoted the deposition of mineralised ECM	^217^
Tendon	Human TDSCs	2	Increased gene expression of tendon-specific marker tenomodulin	^97^
	Human TDSCs	5	Increased gene expression of collagen type I and tenascin C	^98^
Heart	Human vascular-derived myofibroblasts	4–0.5	Increased gene expression of collagen type I and collagen crosslink enzymes lysyl oxidase and lysyl hydroxylase 2	^89^

*ADSCs* adipose-derived stem cells, *BMSCs* bone marrow-derived stem cells, *CCs* chondrocytes, *DFs* dermal fibroblasts, *EKs* epidermal keratinocytes, *TDSCs* tendon-derived stem cells, *VEGF* vascular endothelial growth factor.

Despite the importance of hypoxia in eukaryotic cell culture and in the development of functional cell therapies, routinely cell culture studies are performed under hyperoxic conditions (21% O_2_) that do not match physiological oxygen levels (e.g., 5–13% in blood, 2–9% in most tissues)^105^. Further, hyperoxic cell cultures lead to poor and slow ECM synthesis, cellular senescence and activation of stress pathways^106–108^. Although hypoxia chambers and incubators are utilised to perform hypoxic experiments, implementation of physioxia in industrial scale is expensive to purchase and maintain, thus of limited applicability despite the fact that studies have argued that hypoxia precondition should be a prerequisite for clinical translation of cell therapies^109,110^.

### Mechanical stimulation

Another microenvironmental cue critical for optimal ECM synthesis and deposition is mechanical loading^111^. Considering that uniaxial or multiaxial tensile, compressive or shear mechanical loads regulate ECM composition and function and tissue homoeostasis^112^, mechanical stimulation of tissue-engineered constructs, with the use of bioreactors, is attracting growing interest in order to recapitulate the in vivo microenvironment of native (primarily musculoskeletal) tissues in in vitro setting (Table 8). For example, mechanical stimulation, in the form of shear force, hydrostatic pressure or compression, has been shown to promote ECM synthesis in human chondrocyte and MSC cultures, and to produce tissue‐engineered cartilaginous substitutes^113–115^. Similarly, in tendon engineering, mechanical loading has been shown to maintain tenocyte phenotype and to increase their proliferation and ECM synthesis^116,117^, and to direct MSCs towards tenogenic lineage^118–120^. In bone engineering, mechanical loading has been employed extensively to enhance mineralised matrix synthesis and deposition^121,122^.

**Table 8:** Influence of mechanical stimulation on ECM synthesis and deposition in vitro.

Clinical indication	Cells	Stimulation regime	Outcome	Ref.
Skin	Human DFs	20% strain	Increased collagen type I expression and deposition	^218^
	Human DFs Human EKs	10% strain	Increased deposition of laminin, collagen type IV and collagen type VII in the basal layer of engineered human skin equivalents	^219^
Cartilage	Human BMSCs	10% dynamic compression	Increased collagen type II and aggrecan gene expression	^115^
	Human CCs	50% compression strain	Increased collagen type II, collagen type VI, fibronectin and laminin deposition	^113^
Bone	Human BMSCs	3% strain	Cyclic mechanical strain increased alkaline phosphate activity and mineralised matrix deposition	^220^
	Human BMSCs	10% strain	Cyclic mechanical stimulation increased expression of alkaline phosphatase, osteocalcin and collagen type I, and deposition of mineralised ECM	^221^
Tendon	Human tenocytes	10% strain	Increased synthesis and deposition of collagen type I and gene expression of tendon-specific marker scleraxis	^117^
	Human BMSCs	1.5 mm amplitude	Cyclic loading increased collagen type I and scleraxis expression	^120^
Heart	Human heart cells	20% strain	Increased deposition of a highly organised collagen matrix	^222^

*ADSCs* adipose-derived stem cells, *BMSCs* bone marrow-derived stem cells, *CCs* chondrocytes, *DFs* dermal fibroblasts, EKs epidermal keratinocytes.

With regards to scaffold-free tissue engineering, unidirectional stretching has been applied to induce the alignment of human iPSCs-derived cardiomyocyte sheets after detachment from temperature-responsive dishes^43^. Two weeks after transplantation into the superficial gluteal muscle of athymic rats, the stretched cell sheets retained the unidirectionality of their myocardial fibres, holding great potential for heart engineering. While the application of mechanical loading has not been applied extensively in temperature-responsive systems, it has been successfully implemented in other scaffold-free tissue-engineered models. For example, cartilage constructs have been produced using high-density porcine chondrocytes, centrifugation and non-adhesive agarose substrates. Simultaneous application of cyclic unconfined compression and perfusion to the cartilage constructs increased the deposition of glycosaminoglycans and collagen type II in comparison to static control groups^123^. Another study reported the fabrication of scaffold-free cartilage by applying high-amplitude compressive strain, using porcine chondrocyte seeded onto a hydroxyapatite carrier. Compression amplitude of 20% had the highest positive effect by inducing the synthesis of cartilage-specific ECM and enhancing the mechanical properties of the constructs^124^.

An overwhelming amount of literature has demonstrated the positive effects of mechanical stimulation and/or conditioning on advanced tissue-engineered constructs. Nevertheless, the limited fundamental understanding of the molecular and cellular mechanisms, the lack of standardised protocols for mechanical stimulation and the very high costs of bioreactor systems, limit their scalability and use in commercial space.

### Macromolecular crowding

Although various in vitro microenvironment modulators have been assessed over the years to control cell fate during in vitro culture, only marginally enhance and accelerate ECM synthesis and deposition. For example, mechanical stimulation and oxygen tension have been shown to increase by 2–5-fold ECM synthesis, and deposition both in permanently differentiated and stem cell cultures^125–128^, which although mathematically may be considered as an improvement, commercially, the associated expenditure does not justify the change in the process.

**Fig. 3:**
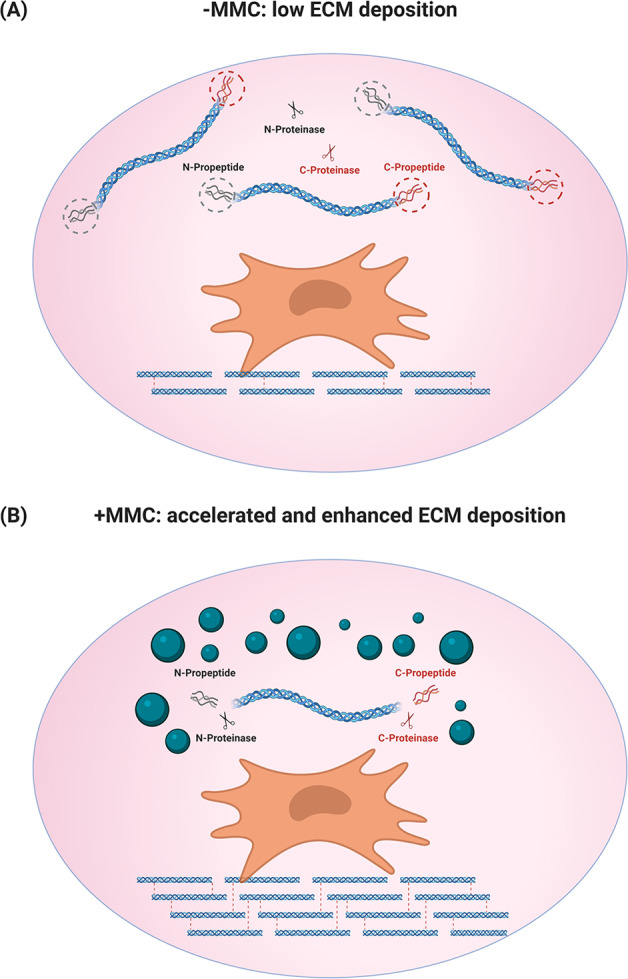
Macromolecular crowding in cell culture. In the dilute cell culture context (−MMC), the N- and C-proteinases and the water-soluble procollagen are diffused, and respectively deactivated and dissolved, resulting in very low amounts of deposited ECM (**A**). Under macromolecular crowding conditions (+MMC), the diffusion of procollagen and N- and C-proteinases is restricted, resulting in enhanced and accelerated ECM deposition (**B**). The figure was created with BioRender.com.

In recent years, macromolecular crowding (MMC) has emerged as a means to substantially increase and accelerate ECM deposition in vitro (e.g., up to 120-fold increase in collagen and associated ECM deposition within 4–6 days in differentiated^129–133^ and stem^134–141^ cell cultures). In tissues, the presence of numerous macromolecules, such as carbohydrates, proteins, lipids and nucleic acids, creates a crowded or confined microenvironment that affects the rate of biological and biochemical reactions^142,143^. The MMC’s mechanism of action is based on the theory of mutual excluded volume effect, which refers to the volume that is inaccessible in the system to new molecules as a result of pre-existing molecules^144^; two molecules cannot be at the same place at the same time. Although the effect of MMC on protein folding and assembly^145–147^, DNA condensation and replication^148–151^ and biochemical reactions^152,153^ has been well established, it is still under investigation in cell culture context. Nonetheless, it is accepted now that in eukaryotic cell culture scenario, MMC accelerates the enzymatic processing of procollagen to collagen, resulting in enhanced collagen, and bound ECM, deposition (Fig. 3). Indeed, in standard cell culture setting, the conversion of water-soluble procollagen to insoluble collagen is relatively slow, since the proteinases required for the enzymatic cleavage of procollagen are dispersed in the dilute culture media. The addition of macromolecules to the culture media results in a more efficient volume occupancy, preventing their dispersion^154^. MMC has been shown to drive the molecular assembly of collagen fibrils in vitro and to stabilise the formed matrix through enzymatic crosslinking^155,156^.

Several macromolecules, alone or in cocktail form, have been utilised as crowders to enhance and accelerate ECM deposition (Table 9). It has been demonstrated that negative charge and polydispersity are key regulators for ECM deposition^129^. Indeed, negatively charged crowders cause a stronger volume-excluding effect due to electrostatic repulsion^157^ and polydispersity, indicative of the heterogeneity of sizes and/or shapes of molecules in a mixture, maximises the excluded volume effect through reduction in diffusion^158^. These prompted the use mixed crowding molecules systems to achieve higher volume exclusion effect, reduced procollagen/proteinases diffusion and ultimately enhanced and accelerated ECM deposition. For instance, a cocktail of Ficoll™ 70 kDa/Ficoll™ 400 kDa/Ficoll™ 1000 kDa has been shown to lead to higher ECM deposition in dermal fibroblast cultures than the traditionally used Ficoll™ 70 kDa/Ficoll™ 400 kDa cocktail^129^. Carrageenan, a naturally polydisperse and negatively sulfated polysaccharide has been shown to induce the highest volume exclusion effect, as judged by the highest and fastest ECM deposition in vitro^136^^,^^159^^,^^160^. Although in traditional protein assembly investigations, for simplicity purposes, crowders are considered as inert macromolecules, eukaryotic cell culture experiments indicate that the chemistry of the crowder affects cell phenotype. For example, in corneal fibroblasts, dextran sulfate-induced myofibroblast trans-differentiation, while the Ficoll™ 70 kDa/400 kDa cocktail^161^ and carrageenan^160^ maintained their phenotype. Further, in stem cell cultures, non-sulfated polysaccharides have been shown to induce adipogenesis^135,137,162^, while sulfated polysaccharides have been shown to induce chondrogenesis and osteogenesis^134,136,140^.

**Table 9:** Macromolecular crowders used in vitro for human cell culture.

Macromolecular crowder	Cell type	Outcome	Refs.
Carrageenan	WS-1, WI-38, osteoblasts, corneal fibroblasts, adult DFs, neonatal DFs, BMSCs, ADSCs, CCs	Increased ECM deposition modulated by crowder polydispersity, enhance chondrogenesis and osteogenesis of MSCs, development of ECM-rich tendon equivalents, deposition of fibrocartilage matrix in CCs	^129^^,^^130^^,^^134^^,^^136^^,^^140^^,^^159^^,^^160^^,^^163^^,^^164^^[,175,176,223^
Dextran sulfate 10 kDa	WI-38, DFs	Accelerated conversion of procollagen to collagen in WI-38, but not in DFs	^129,132^
Dextran sulfate 100 kDa	DFs	Accelerated conversion of procollagen to collagen	^129^
Dextran sulfate 500 kDa	WS-1, WI-38, corneal, dermal and hypertrophic scar fibroblasts	Increased ECM deposition, myofibroblastic differentiation of corneal fibroblasts	^132^^,^^156^^,^^163^^,^^164^^,^^171^^[,224^
Dextran sulfate 670 kDa	WI-38	Increased ECM deposition	^156^
Ficoll^™^ 70 kDa	WI-38 fibroblasts, DFs	No increase in ECM deposition	^129,132^
Ficoll^™^ 400 kDa	WI-38 fibroblasts, DFs	No increase in ECM deposition	^129,132^
Ficoll^™^ 1000 kDa	DFs	No increase in ECM deposition	^129^
Ficoll^™^ 70 kDa and 400 kDa cocktail	WI-38, WS-1, corneal, dermal and vocal cord fibroblasts, keratinocytes, BMSCs, ADSCs	Increased ECM deposition, increased deposition of collagen IV and perlecan, enhanced adipogenesis, increased deposition of total collagens and glycosaminoglycans	^129^^,^^130^^,^^136^^,^^137^^,^^139^^,^^141^^,^^163^^,^^170^^,^^173^^[,225^
Ficoll^™^ 70 kDa, 400 kDa and 1000 kDa cocktail	DFs	Increased collagen I deposition modulated by crowder polydispersity	^129^
PEG 4 kDa	DFs	Full processing of procollagen to collagen	^226^
Polysodium-4-styrene 200 kDa	WI-38	Accelerated conversion of procollagen to collagen	^132^
Polyvinylpyrrolidone 40 kDa	DFs, BMSCs	Increased ECM deposition	^227^
Polyvinylpyrrolidone 360 kDa	DFs, BMSCs	Increased ECM deposition	^227^
Seaweed polysaccharides (fucoidan, galactofucan, ulvan)	ADSCs	Increased ECM deposition, enhanced osteogenesis and chondrogenesis	^140^

*ADSCs* adipose-derived stem cells, *BMSCs* bone marrow stem cells, *CCs* chondrocytes, *DFs* dermal fibroblasts, *MSCs* mesenchymal stem cells.

In the field of scaffold-free tissue engineering, MMC has advanced the production of ECM-rich cell sheets, showing the possibility to dramatically speed up the production of implantable tissue equivalents^130^^,^^163^^,^^164^. Interestingly, these studies demonstrated that the commercially available pNIPAM-based culture dishes were not able to induce detachment of intact cell sheets, due to the presence of abundant ECM produced under MMC conditions. Co-polymerisation of pNIPAM with the hydrophobic N-tert-butylacrylamide (NTBA) monomer, at an optimal ratio of 35%, allowed for first time the production of dense and cohesive cell sheets with intact cell–cell and cell–ECM junctions. The efficiency of the pNIPAM-NTBA copolymer to produce such ECM-rich construct was attributed to additional steric hindrance induced by the NTBA group, which decreased hydrogen bonding and consequently decreased protein adsorption, ultimately facilitating cell detachment. In addition, MMC has been used for the development of scaffold-free cell-derived matrices^133^^,^^141^^,^^165^ that have been used successfully for in vitro cell propagation^166–168^ and for the generation of skin equivalents with complete stratification and a mature dermal–epidermal junction for either regenerative medicine^169,170^ or drug discovery purposes^171–174^. To further boost the development of scaffold-free tissue equivalents, multifactorial approaches combining MMC and different in vitro microenvironment modulators have been explored. For example, the use of MMC in combination with low oxygen tension synergistically contributed to the development of ECM-rich tissue equivalents^136,159,160,175^. Similarly, the use of MMC simultaneously to mechanical stimulation facilitated the fabrication of tendon-like tissue constructs in vitro^176^.

Despite the significant contribution and potential of MMC in tissue engineering, the optimal (with respect to maximum ECM deposition in the shortest period of time, while precisely controlling cell fate) crowding agent/cocktail remains elusive.

## Roadmap to commercialisation

Although significant strides that have been achieved in the production of ECM-rich tissue equivalents within commercially relevant timeframes, a major roadblock in the commercialisation of cell-based strategies is the high costs of manufacturing solutions, which need to comply with current good manufacturing practices. A recent study analysed eight case studies in Europe using both autologous and allogeneic therapies (academic and other small-scale enterprises scale) and estimated manufacturing costs (i.e., materials, equipment, personnel and facility running costs) to be in the range of € 23,033 and € 190,799 per batch, with batch yield varying between 1 and 88 doses^177^. With regards to scaffold-based strategies, another study reported the costs of stem cell-engineered airway transplants to range from US$ 174,420 to US$ 740,500 in three UK patients^178^. It is worth noting that such regenerative medicine strategies are also associated with several risks (e.g., human errors, batch-to-batch variability, high risk of contamination), which further limit their availability^179^. In order to overcome these limitations, automated platforms could significantly reduce the cost of goods up to 30% (ref. ^180^), ensuring reliability and reproducibility across the life cycle of the product, from cell isolation and expansion to in‐line product quality assurance^181,182^. To further standardise the manufacturing process, the development of xeno-free, chemically defined media has been advocated to reduce the risk of pathogen sources and simplify the regulatory approval^183,184^.

Besides reducing the costs associated to manufacturing systems, it is also imperative to consider from outset both regulatory hurdles and reimbursement concepts to successfully translate cell-based concepts into blockbuster therapies. In fact, one should consider that no Advanced Therapy Medicinal Products (ATMPs) has yet achieved widespread reimbursement and access across the five biggest European countries (Germany, France, UK, Italy, Spain), and many have been restricted beyond the regulatory label^185^. Both public and private healthcare systems are still under-equipped to absorb the high financial implications associated with cell therapies, also considering additional costs related to expensive hospital stays, procedures and rehabilitation, which come on top of the product price. Another factor which limits cellular therapies to secure reimbursement following market authorisation is the lack of comparative effectiveness data. Manufacturers must demonstrate that the product has incremental benefit against existing standard of care, not only from an economical perspective, but, most importantly, from a clinical one, ensuring long-term safety and efficacy^186^. For example, ChondroCelect^®^, an autologous chondrocyte therapy licensed for the treatment of single symptomatic cartilage defects of the femoral condyle of the knee, despite being the first cell-based regenerative therapy to obtain a centralised marketing authorisation in Europe, has been withdrawn from the market, failing to established robust clinical efficacy and consequently not fulfilling reimbursement criteria^187^.

Considering the unpredictability around the long-term efficacy and safety of regenerative medicines, new models for financing and reimbursement have been proposed to ensure patient access to such therapies. For instance, annuity or instalment payment models minimise high up-front single payment, allowing healthcare providers to amortise the cost of therapies over multiple years, and recognises the potential of single-administered cell therapies based on evidence that the treatment continues to be effective over a specified period of time^188^. Another example are pay-for-performance models, where the reimbursement for a treatment depends on whether a specified clinical outcome is achieved. These models aim to partially shift financial risks from payers to manufacturers, which may prompt the interest of healthcare providers^189^.

Overall, cell manufacturers need to address many challenges during product development, in order to gain approval from regulatory authorities and ensure that the proposed therapy will not be held back by reimbursement policies.

## Conclusions

In vitro scaffold-free organogenesis has come long way since early 1980’s that was first appeared in the literature. Despite though the significant strides in in vitro, preclinical and clinical setting, only a handful of concepts have become clinical and commercial reality. Issues associated with required number of functional cells, dimensionality, production timeframe, automation, scalability (that affect reimbursement) and regulatory requirements/classification must be addressed for wide acceptance of this transformative and disruptive concept.
